# Metabolic rewiring directs melanoma immunology

**DOI:** 10.3389/fimmu.2022.909580

**Published:** 2022-08-08

**Authors:** Ningyue Sun, Yangzi Tian, Yuhan Chen, Weinan Guo, Chunying Li

**Affiliations:** ^1^ Department of Dermatology, Xijing Hospital, Fourth Military Medical University, Xi’an, China; ^2^ School of Basic Medical Sciences, Fourth Military Medical University, Xi’an, China

**Keywords:** melanoma, metabolism, immunology, immunotherapy, glycolysis

## Abstract

Melanoma results from the malignant transformation of melanocytes and accounts for the most lethal type of skin cancers. In the pathogenesis of melanoma, disordered metabolism is a hallmark characteristic with multiple metabolic paradigms involved in, e.g., glycolysis, lipid metabolism, amino acid metabolism, oxidative phosphorylation, and autophagy. Under the driving forces of oncogenic mutations, melanoma metabolism is rewired to provide not only building bricks for macromolecule synthesis and sufficient energy for rapid proliferation and metastasis but also various metabolic intermediates for signal pathway transduction. Of note, metabolic alterations in tumor orchestrate tumor immunology by affecting the functions of surrounding immune cells, thereby interfering with their antitumor capacity, in addition to the direct influence on tumor cell intrinsic biological activities. In this review, we first introduced the epidemiology, clinical characteristics, and treatment proceedings of melanoma. Then, the components of the tumor microenvironment, especially different populations of immune cells and their roles in antitumor immunity, were reviewed. Sequentially, how metabolic rewiring contributes to tumor cell malignant behaviors in melanoma pathogenesis was discussed. Following this, the proceedings of metabolism- and metabolic intermediate-regulated tumor immunology were comprehensively dissertated. Finally, we summarized currently available drugs that can be employed to target metabolism to intervene tumor immunology and modulate immunotherapy.

## Background

Melanoma is the deadliest type of skin cancer caused by the malignant transformation of melanocytes. In 2021, there were estimated 106,110 new cases emerging and 7,180 deaths due to melanoma in the United States (1). As melanocytes are widely distributed in organs and tissues such as uvea, mucosa, inner ear, and rectum in addition to the skin epidermis, melanoma arising in different anatomical locations is accordingly categorized into four subtypes, namely, cutaneous melanoma (CM), acral melanoma (AM), uveal melanoma (UM), and mucosal melanoma (MM). CM is of a rather high mutation rate (2), especially in certain genes like those involved in the mitogen-activated protein kinase (MAPK) pathway, such as v-raf murine sarcoma viral oncogene homolog B (*BRAF*) and ras viral (V-Ras) oncogene homolog (*RAS*) (3, 4). Accordingly, BRAF inhibitors vemurafenib, dabrafenib, and encorafenib, as well as mitogen-activated protein kinase kinase (MEK) inhibitor trametinib, cobimetinib, and binimetinib, are applied to the targeted treatment of melanoma (5, 6). Although demonstrating better clinical efficacy than conventional chemotherapies (7, 8), these BRAF and MEK inhibitors engender inevitable drug resistance caused by complex mechanisms, which eventually lead to tumor cell proliferation and poor prognosis of melanoma patients (9–11).

Previous investigations of melanoma pathogenesis and therapeutic approaches generally focused on the malignant behaviors of tumor cells. While the study of the tumor microenvironment (TME) has been extensive in recent years, therapeutic strategies directly targeting the TME have not been clinically adopted. The TME is composed of mixed populations of cells such as melanoma cells and surrounding immune cells, cancer-associated fibroblasts (CAFs), and keratinocytes (12). Tumor-infiltrating immune cells gradually exhibit malfunction in the dynamic interplay with melanoma cells and finally become “accomplices” of melanoma immune escape. Immune checkpoints prominently underlie the dysfunction of immune cells; thus, the blockades of immune checkpoints programmed cell death protein 1 (PD-1) (nivolumab and pembrolizumab), programmed cell death ligand 1 (PD-L1) (atezolizumab), and cytotoxic T lymphocyte antigen 4 (CTLA-4) (ipilimumab) are applied in melanoma immunotherapy. Encouragingly, the combination of nivolumab (anti-PD-1 antibody) and ipilimumab (anti-CTLA-4 antibody) has been documented to improve the overall 5-year-survival of melanoma patients to 52% with a response rate of 58% (13), which underscores the importance of the interactions between melanoma cells and immune cells in the TME.

The interruptions of antitumor immunity in the TME partly stem from melanoma metabolic reprogramming. Multiple fundamental metabolic paradigms such as glycolysis, oxidative phosphorylation, amino acid metabolism, autophagy, and lipid metabolism are rewired by oncogenic factors in melanoma (14–17), which not only provide sufficient energy and building bricks of macromolecules biogenesis but also manipulate the TME to help melanoma immune evasion. For instance, enhanced glycolysis of tumor cell leads to the acidification of the TME, which is associated with mitigated infiltration and antitumor function of natural killer (NK) cells and CD8^+^T cells, as well as the resistance to immunotherapy (18–20). However, activated lipid metabolism in tumor potentiates melanoma immunogenicity and thereby increases its sensitivity to T cell-mediated killing, rendering a higher response rate to anti-PD-1 immunotherapy (21). Apart from tumor cell metabolism, the dysregulated metabolism in immune cells also undermines their antitumor capacity. For example, peroxisome proliferator-activated receptor gamma, coactivator 1 alpha (PGC-1α) upregulation in CD8^+^T cells can favor the central memory formation and provide stronger antitumor capacity in restraining melanoma progression in a preclinical mouse model (22). Therefore, dysregulated metabolism in both tumor cells and immune cells affects antitumor immunity, which underpins the significance of illustrating the implication of metabolism in immunologic characteristics of the TME in melanoma and the role of targeting metabolism in modulating the efficacy of immunotherapy.

## The tumor microenvironment and antitumor immunity

The antitumor capacity of immune cells in the TME is dynamically remodeled in melanoma. During the early stage of tumor progression, tumor-infiltrating immune cells can fulfill their responsibility to detect and eradicate tumor cells effectively *via* the cooperation of innate and adaptive immunity. To be specific, once the infiltrating natural killer (NK) cells detect and exert a killing effect on melanoma cells, they concurrently secrete cytokines to recruit antigen-presenting cells (APCs), especially dendric cells (DCs), to engulf the dead tumor cells or process tumor cells into damage-associated molecular patterns (DAMPs) (23). Hereafter, DCs gradually mature and migrate to lymph nodes, where they process and load cancer antigens onto human leukocyte antigen class-1 (HLA-1) for presentation to CD8^+^T cells, meanwhile upregulating costimulatory molecule expression and pro-inflammatory cytokine secretion to activate naive T cells (24). Thereby, melanoma-specific effector T cells are recruited to the TME to take their responsibility for the eradication of melanoma cells. In this immune elimination process, NK cells act as the frontline of defense and the main component of antitumor innate immunity. Actually, the response of NK cells to target cells depends on the balance between activating signals and inhibitory signals mediated by surface receptors. Inhibitory receptors are represented mainly by HLA class I-binding receptors (KIR, NKG2A, and LIR-1/ILT2), while activating receptors include NKp46, NKp30, NKp44, NKG2D, and DNAM-1. The susceptibility of tumor cells to NK cells would largely depend on the high expression levels of activating receptor–ligands, but it can be further increased by the downregulation of HLA class I expression in melanoma cells (25). Besides, they exert an immunomodulatory effect through the production of cytokines such as interferon-γ (IFN-γ) and tumor necrosis factor-α (TNF-α) and contribute to the recruitment and maturation of APCs through secreting chemokines like CC chemokine ligand 3 (CCL3), CCL4, and CCL5, which provide a linkage between innate and adaptive immunity (26). APCs not only ingest and present tumor antigens but also upregulate the expression of a large variety of co-stimulatory receptors on their surfaces, like CD80 and CD86, which are pivotal for T-cell priming (27). Besides, several chemotactic receptors are also upregulated on APCs, for instance, CCR7 (CD197), which induces the migration to lymph nodes for antigen presentation and activation of T cells (28, 29). As for the adaptive immunity, CD8^+^T cells and CD4^+^T cells can be properly activated *via* two co-stimulatory signals. The first is the combination between T-cell receptor (TCR) and major histocompatibility complex (MHC) molecule (CD8^+^T cells with MHC class I vs. CD4^+^T cells with MHC class II), and the second is the binding of co-stimulation signal molecules present on T cells (e.g., CD28) to their corresponding receptors localized on APCs (e.g., CD80 and CD86) (30). Subsequently, activated CD8^+^T cells and CD4^+^T cells are recruited to the TME by the chemokine gradient of CXCL9, CXCL10, and CXCL11 generated by DCs and tumor-associated stroma (31). Then, melanoma cells expressing a high level of costimulatory molecules directly present a tumor-associated antigen (TAA)–MHC compound to CD8^+^T cells, stimulating the production of interleukin 2 (IL-2) that leads to their proliferation and differentiation to cytotoxic T cells (CTL). CTLs bind to melanoma cells *via* TAA presented on MHC molecules and then exert an antitumor effect on melanoma cells by releasing perforin and granzyme B to induce apoptosis and secreting cytokines such as IFN-γ and TNF to reinforce the inflammatory TME. Meanwhile, CD4^+^T cells can differentiate into several types of functional cells depending on the cytokines in TME (32, 33). When diverse T cells cooperatively kill melanoma cells, more TAA can be released to facilitate antitumor immunity. Another kind of cells involved in antitumor defense is macrophages. The pro-inflammatory subtype of macrophage M1 can be activated by Th1 cells and pro-inflammatory factors such as IL-6, IL-12, IL-23, and TNF-α. Upon activation, M1 can exert non-specific killing through secreting TNF-α and performing non-specific phagocytosis and exerting an antibody-dependent cell-mediated cytotoxicity (ADCC) effect. Also, they can act as antigen-presenting cells and enhance adaptive immunity. Yet the other subtype of macrophages, M2, is generally activated by Th2 cells and anti-inflammatory stimuli such as IL-4, IL-10, and IL-13, resulting in anti-inflammatory and pro-tumorigenic effects (34, 35).

However, the surveillant and self-defensive efficacy of tumor-infiltrating immune cells is dampened in the cancer-immunoediting process during the equilibrium phase. In this phase, melanoma cells reshape their immunologic features when in interactions with immune cells, including the downregulation of tumor-associated antigens (TAAs) and MHC molecules; the deletion in antigen-processing machinery; the upregulation of immune checkpoints PD-L1, TIM-3, and LAG-3; and the secretion of chemokines and cytokines, and finally result in melanoma immune escape (36, 37). Initially, melanoma mutants featuring downregulated TAA and MHC molecule expressions are reserved in the “natural selection” by the TME, rendering these melanoma cells invisible to antitumor immunity (38). Further, melanoma cells also alter the functions of various infiltrating immune cells in the TME, especially T lymphocytes, NK cells, DCs, and macrophages, to facilitate immune escape.

The antitumor effects of melanoma-specific CD8^+^T cells are inhibited by aberrantly expressed immune checkpoints on the surface of tumor cells. Chronic exposure to tumor antigens and the overexpression of CTLA-4 and PD-L1 on melanoma cells raise the inhibitory receptors on T cells, which hinder their activation. Besides, inhibitory molecules, such as regulatory T cell (Treg)-derived TGF-β, restrain the cytotoxic capability of CTLs. Indoleamine 2,3-dioxygenase 1 (IDO1) deprives CD8^+^ T and DCs of tryptophan needed for their metabolism, resulting in deficiency of T-cell activation and antitumor function (39). While T cells mainly contribute to adaptive antitumor immunity, NK cells are responsible for innate immunity for tumor control. Functional NK cells are able to detect and eradicate melanoma cells with a low MHC-I expression, which are unable to be recognized by cytotoxic T cells, yet they are also silenced by suppressive molecules. In the interaction with NK cells, melanoma cells express increased prostaglandin E2 (PGE2) and IDO, leading to the shrinkage of activating receptors on NK cells and thus attenuating NKs’ capability of binding to and killing melanoma cells (25). As the most potent antigen-presenting cells in the immune system, DCs are also enervated by certain suppressive molecules, i.e., TGF-β, IDO, IL-2, and vascular endothelial-derived growth factor (VEGF), and are even induced to premature apoptosis for expressing apoptotic molecules (40–42).

In contrast to inhibited antitumoral immune cells, pro-tumoral immune cells are boosted in the stage of immune escape. Regulatory T cells are the immunosuppressive cells preventing the overreaction of the immune system through expressing CTLA-4 and secreting immune-suppressive molecules. However, it is observed that in melanoma TME, Tregs are abnormally activated and thus suppress the activity of antitumor immune cells, leading to melanoma immune escape. Other immune cells in the TME including myeloid-derived suppressor cells (MDSCs), B cells, and neutrophils all play their parts in the modulation of antitumor immunity. In addition, cancer-associated fibroblasts (CAFs) and endothelia in the TME also affect angiogenesis and tumor development by remodeling the extracellular matrix, interacting with surrounding cells and secreting soluble growth factors (43–45).

Altogether, multiple cells and various molecules synergistically weave a TME in which tumor cells are restricted initially but then develop and finally escape the immune surveillance. More mechanisms underlying the interactions within the TME remain to be revealed to optimize the diagnoses and treatments of melanoma.

## Proceedings of metabolic dysregulation in melanoma pathogenesis

Multiple paradigms of cellular metabolism such as glycolysis, oxidative phosphorylation, amino acid metabolism, autophagy, and lipid metabolism are altered in melanoma cells to ensure the supplement of sufficient energy and precursors for macromolecular biosynthesis. In melanoma, these metabolic alterations are of relatively high plasticity, which is tightly associated with and greatly implicated in the regulation of tumor cell behavior. Hence, an insight into the proceedings of metabolic dysregulation of melanoma is necessary.

## Reprogramming of glucose metabolism in melanoma

### Glycolysis

Aerobic glycolysis is robustly activated in various types of tumors including melanoma to support their increased energetic and biosynthetic demands. Melanoma cells promote the activation of glycolysis through various mechanisms; thereinto, genetic mutations are at the fundamental place. By profiling metabolites in 17 patient-derived xenograft melanoma models, Shi et al. identified that compared with *BRAF*-wild-type tumors, *BRAF*-mutant tumors have the metabolomics and metabolic flux characteristics of enhanced glycolysis (46). Mechanically, *BRAF* mutation directly upregulates a series of transcription factors, glucose transporters, and kinases to promote glycolysis, for instance, hypoxia-inducible factor-1α (HIF-1α), myc proto-oncogene protein (MYC), glucose transportase-1 (Glut 1), Glut 3, and hexokinase 2 (HK2) (47). In addition, the activation of glycolysis in melanoma cells is putatively a compensation for the inhibited mitochondrial oxidative phosphorylation. *BRAF*-activated melanomas are characterized by a suppressed level of microphthalmia-associated transcription factor (MITF) and thus a low PGC-1α expression, which leads to attenuated mitochondrial function (14). Aside from genetic regulation by *BRAF*, the overactivated MAPK pathway in melanoma also contributes to increased glycolysis. Ribosomal S6 kinase 1 (RSK), the substrate of extracellular signal-regulated kinase 1/2 (ERK1/2) in the MAPK pathway, directly phosphorylates and activates fructose-6-phosphate 2-kinase/fructose-2,6-bisphosphatase 2 (PFKFB2), an enzyme catalyzing the synthesis of fructose-2,6-bisphosphatase, to promote melanoma glycolysis (48). Enhanced glycolysis provides melanoma cells with abundant energy and metabolic intermediates, promoting the synthesis of proteins, nucleic acids, fatty acids, and other macromolecules needed for further growth and proliferation, while inhibiting the entire glycolysis process by targeting the *BRAF* or *MAPK* pathway may help hamper the growth and spread of melanoma (49).

### Mitochondrial oxidative phosphorylation

Mitochondrial oxidative phosphorylation (OXPHOS) also provides energy, but it is generally thought to be inhibited in tumor cells due to the canonical Warburg effect, which leads to the omittance of its significant role in melanoma survival and growth. However, PGC1α-positive melanoma cells show better capability of energy generation, resistance to reactive oxygen species (ROS), and thus improved viability in hypoxia conditions than PGC1α-negative ones (50). In addition, since *BRAF* suppresses mitochondrial function *via* the downregulation of MITF, melanoma cells treated by BRAF-targeted drugs restore oxidative phosphorylation to maintain their survival, leading to adaptive resistance to targeted therapy (51). Moreover, it has been documented that long non-coding RNA (lncRNA) SAMMSON, which co-expresses with MITF, promotes melanoma growth by upregulating mitochondrial main regulatory factor p32 and targeting SAMMSON renders melanoma cells therapeutic vulnerability (52, 53). However, while PGC1α upregulates OXPHOS to provide energy for melanoma cells, it suppresses metastasis. PGC1α enhances the transcription of inhibitor of DNA binding 2 (ID2), which in turn binds to and inactivates the transcription factor 4 (TCF4), leading to the downregulated expression of metastasis-related genes and thus impeding melanoma metastasis (54). In contrast to this report, another two investigations have demonstrated that the suppression of mitochondrial respiration function can robustly restrain the invasive capacity and metastasis of melanoma cells (55, 56). Conclusively, the role of mitochondrial function in melanoma metastasis remains controversial and needs further investigations to elucidate the discrepancy. Moreover, oxidative phosphorylation seems to be a promising target to overcome the adaptive resistance to targeted therapy, which is of considerable translational potential in future clinical practice.

## Reprogramming of amino acid metabolism in melanoma

The metabolisms of different types of amino acid, especially glutamine, serine, cystine, and branched-chain amino acids, are ubiquitously aberrant in melanoma cells. In some subtypes of melanoma cells, glutamine metabolism is enhanced *via* c-myc-mediated upregulation of glutaminase (15). This hyper-activated metabolism of glutamine, an anaplerotic substrate, replenishes carbon and nitrogen for almost every melanoma cell metabolic activity and promotes melanoma growth (57). In addition, glutamine compensates for the energy deficiency triggered by the therapeutic dual suppression of glycolysis and mitochondrial oxidative phosphorylation, which leads to drug resistance (58). Of note, while glutamine dependence underlies the resistance to the *BRAF* inhibitor (59), local deprivation of glutamine in melanoma TME increases histone hyper-methylation, which contributes to tumor cell dedifferentiation and also results in resistance to BRAF inhibitor treatment (60). Therefore, the role of glutamine metabolism in drug resistance toward *BRAF* inhibitors remains under debate. Apart from glutamine metabolism, the synthesis of serine is also significantly increased in melanoma cells (61). The upregulation of serine through dietary serine supplementation or gene overexpression of 3-phosphoglycerate dehydrogenase (PHGDH), the rate-limiting enzyme in serine synthesis, effectively promotes melanoma development (62). In a serine and glycine-limited brain environment, melanoma is highly dependent on serine synthesis for brain metastasis. Targeting PHGDH, which catalyzes the rate-limiting step of glucose-derived serine synthesis, is promising in restraining melanoma brain metastasis (63). Furthermore, the upregulation of PHGDH expression contributes to the resistance to MAPK-inhibiting therapy (64). Another significantly-reprogrammed type of amino acid is cystine. The expression of *SLC7A11*, which encodes the cystine/glutamate antiporter Xc^-^ to mediate cystine uptake, is significantly higher in melanoma tissues compared to the controls. The increased cystine uptake elevates the intracellular cysteine level to facilitate the synthesis of glutathione (GSH) and improve the antioxidant capability and viability of melanoma cells (65). In addition to the abovementioned glutamine, serine, and cystine, the metabolism of branched-chain amino acids (BCAAs) also demonstrates pathogenic significance in melanoma. The three BCAAs, namely, leucine (Leu), isoleucine (Ile), and valine (Val), share similar chemical properties and metabolic pathways and provide carbon and nitrogen for biosynthesis and energy generation for melanoma cells. Branched-chain amino acid transaminase 1 and 2 (BCAT1/2) plays a pivotal role of transferring the nitrogen of BCAAs to α-ketoglutarate (α-KG) to produce glutamine and the specified branched-chain keto acid (BCKA) for further catabolism (66). The expression of BCAT1 is prominently upregulated in melanoma cells, and the knockdown of BCAT1 suppresses tumor growth through the suppression of oxidative phosphorylation (67). Moreover, it is discovered that melanoma cells with *BRAF* mutation are highly dependent on leucine, the deprivation of which results in defective autophagy in tumor cells and thus reveals a targetable liability (68). In aggregate, the dysregulation of amino acid metabolism serves as a motivator of the progression of melanoma.

## Reprogramming of autophagy in melanoma

**Figure 1:**
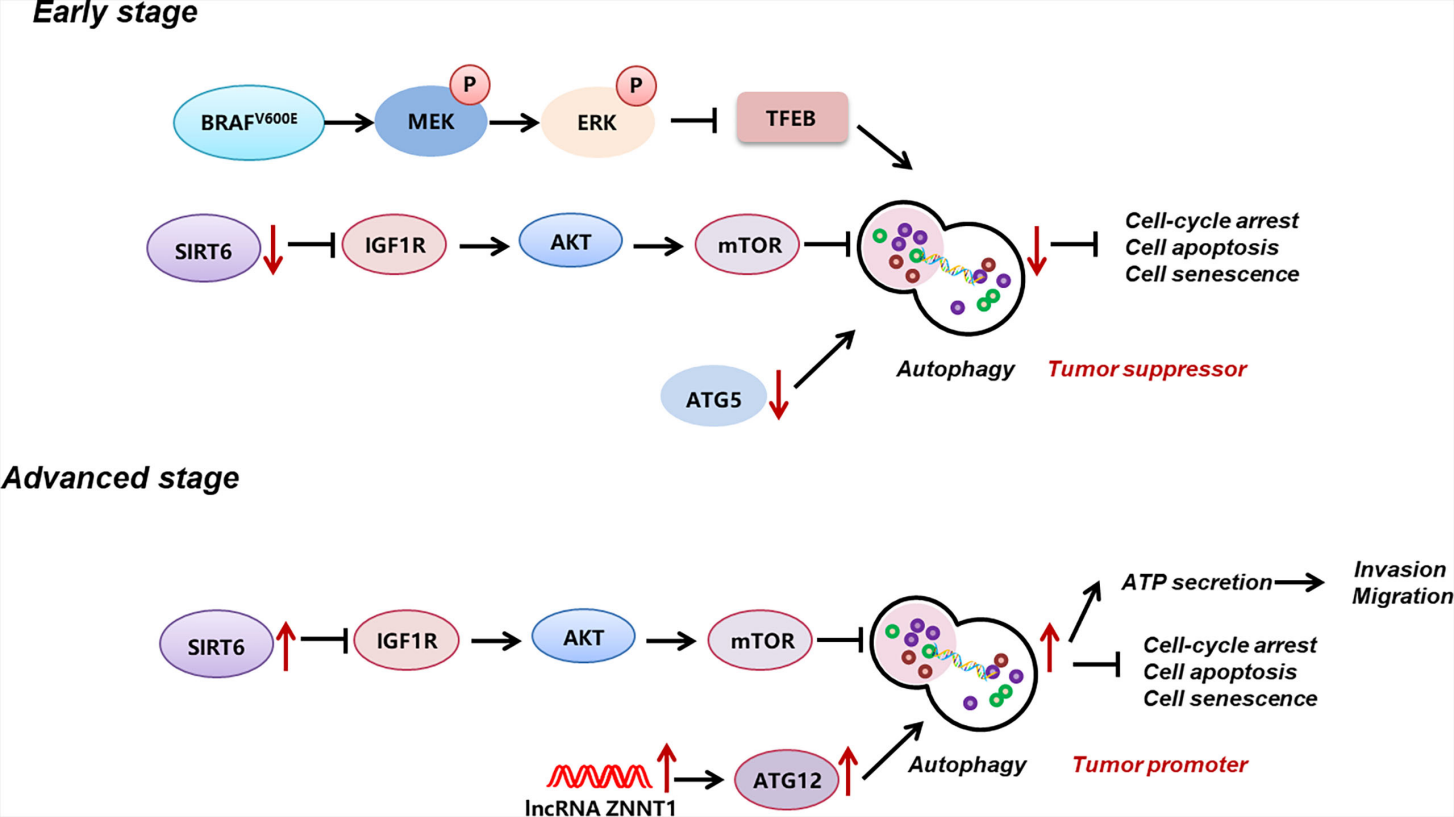
Reprogramming of autophagy in melanoma. During early stage, autophagy level in melanoma is significantly down-regulated, which is induced by MAPK activation-induced suppression of TFEB, the down-regulation of ATG5, and the down-regulation of SIRT6 and its-mediated IGF1R-AKT signaling. During advanced stage, autophagy level is prominent increased, which is related to the up-regulation of SIRT6 and its-mediated IGF1R-AKT signaling, and the up-regulation of lncRNA ZNNT1 and ATG12. Autophagy plays a bimodal role in melanoma progression, namely, acts as a tumor suppressor at early stage, whereas acts as a tumor promoter at advanced stage. .

Autophagy is a pivotal cellular degradation process in which intracellular proteins and organelles were digested in lysosomes to respond to diverse-cell stress and maintain cellular homeostasis. In melanoma, the histone deacetylase sirtuin 6 (SIRT6)-mediated epigenetic modulation of the insulin-like growth factor 1 receptor (IGF1R)-protein kinase B (AKT) pathway results in the downregulation of autophagy in the early stage and an upregulation in the metastatic stage (16). In addition, the canonical melanoma mutation *BRAF* ^V600E^ inhibits autophagy by phosphorylating and inactivating transcription factor EB (TFEB), the master transcriptional factor of autophagy, *via* downstream ERK (69). Dysregulated autophagy in melanoma plays paradoxical roles in melanoma progression. On the one hand, enhanced autophagy mediates the extracellular ATP secretion, which confers the invasion and migration of melanoma cells as well as drug resistance to BRAF-targeted therapy (70). Moreover, melanoma autophagy suppression due to autophagy-related-7 (Atg7) deficiency in a *BRAF* ^V600E^-mutant, phosphatase and tensin homolog (*Pte*n)-null mouse model induces oxidative stress and senescence of melanoma cells and ultimately impedes melanoma development (71). On the other, in *BRAF* ^V600E^ mice wild-type for *Pten*, the previously mentioned senescence obstacle ATG7 inversely promotes melanoma senescence and thus acts as a barrier to melanoma occurrence and development (71, 72). Another critical autophagy regulator ATG5 is documented to be downregulated in the early stage of melanoma, enabling the bypass of BRAF-induced senescence and contributing to the proliferation of melanoma cells (73, 74). Besides, the upregulation of lncRNA ZNNT1 increases the expression of ATG12 to promote autophagy, leading to inhibition of tumorigenesis and migration of UM cells (75). These reports have contrarily demonstrated the suppressive role of autophagy in melanoma progression. Later on, Wang et al. discovered that, in the early stage of melanoma, the autophagy level is significantly downregulated and plays a role of tumor suppressor, while in the metastatic stage, its level is prominently increased to facilitate tumor progression. This study provides a relatively rational explanation for the paradoxical role of autophagy in different stages of melanoma (16) (**Figure 1**). Apart from its dual function in melanoma development, autophagy activation underlies drug resistance to BRAF-targeted therapy, whereas its inhibition improves the therapeutic effect (76, 77). Similarly, targeting GNAQ/11 activates the MAPK pathway which induces melanoma autophagy, resulting in therapy ineffectiveness in UM treatment, and the concomitant inhibition of Gα and autophagy increases melanoma cell death and prolongs the survival of mice bearing melanomas (78). These findings hint that the modulation of autophagy may serve as a possible approach for melanoma treatment.

### Reprogramming of lipid metabolism in melanoma

Lipid metabolism which provides energy and substances for melanoma survival and proliferation is also altered in melanoma (79). Melanoma cells with high invasive capability rely on phospholipids for biofilm synthesis and fatty acids (FAs) for ATP synthesis. Therefore, advanced melanomas are highly dependent on the increased uptake of exogenous lipids, which is another hallmark of melanoma (80). Zhang et al. have proved that in transgenic zebrafish and xenograft mice, melanoma cells adjacent to stromal adipocytes can intake adipocyte-derived lipid through the overexpressed fatty acid transport protein 1 (FATP1/SLC27A1) lipid transporter. This melanocyte-specific FATP1 synergizes with *BRAF* ^V600E^ in promoting the proliferation of cancer cells, and its pharmacological inhibition causes tumor regression (81). In addition to acquiring lipids from adjacent cells, melanoma cells can absorb dietary lipids by expressing high-level CD36, a membrane-bound exogenous lipid deliverer, to facilitate their metastasis. The inhibition of CD36 restricts melanoma metastasis and improves prognosis of melanoma patients (17). In addition to intaking more exogenous lipids, *de novo* lipogenesis is activated in melanoma cells through upregulating the enzymes controlling lipid synthesis—ATP-citrate lyase (ACLY) and sterol regulatory element-binding protein (SREBP), for instance. The inhibition of these enzymes causes melanoma withdrawal, reaffirming the significance of lipid metabolism in melanoma cells (82, 83). Of note, the activation of lipid-producing enzymes such as ACLY and SREBP1 also causes drug resistance of melanoma toward targeted therapy. ACLY induces the resistance to MAPK inhibition by activating acetyltransferase P300 to acetylate the histone at the MITF locus, thus promoting the transcription of the MITF-PGC1 α axis and ultimately facilitating melanoma growth (84). More importantly, *in vivo* preclinical trials show that the inhibition of SREBP-1 can effectively improve the sensitivity to targeted therapy (85). In sum, the metabolism of lipid is activated to promote melanoma growth through various ways and targeting lipogenesis could be employed as a promising strategy to enhance the effectiveness of targeted therapy.

## The crosstalk between metabolism rewiring and melanoma immunology

In addition to providing abundant nutrients for biosynthesis and energy generation to support melanoma survival and proliferation, metabolic rewiring alters both the immunological characteristics of melanoma cells and the functions of immune cells in the TME, ultimately leading to immune escape and the impaired efficacy of immunotherapies.

## The crosstalk between aerobic glycolysis and tumor immunology in melanoma

Aerobic glycolysis is among the earliest revealed metabolic hallmarks of cancer, which alters the immune profile of melanoma cells and the antitumor immune function of immune cells. Thus, elucidating the network of glycolysis-related melanoma immunology is of great significance in understanding the immunological behaviors of melanoma cells.

### Glycolysis in immunologic characteristics of melanoma cells

**Figure 2:**
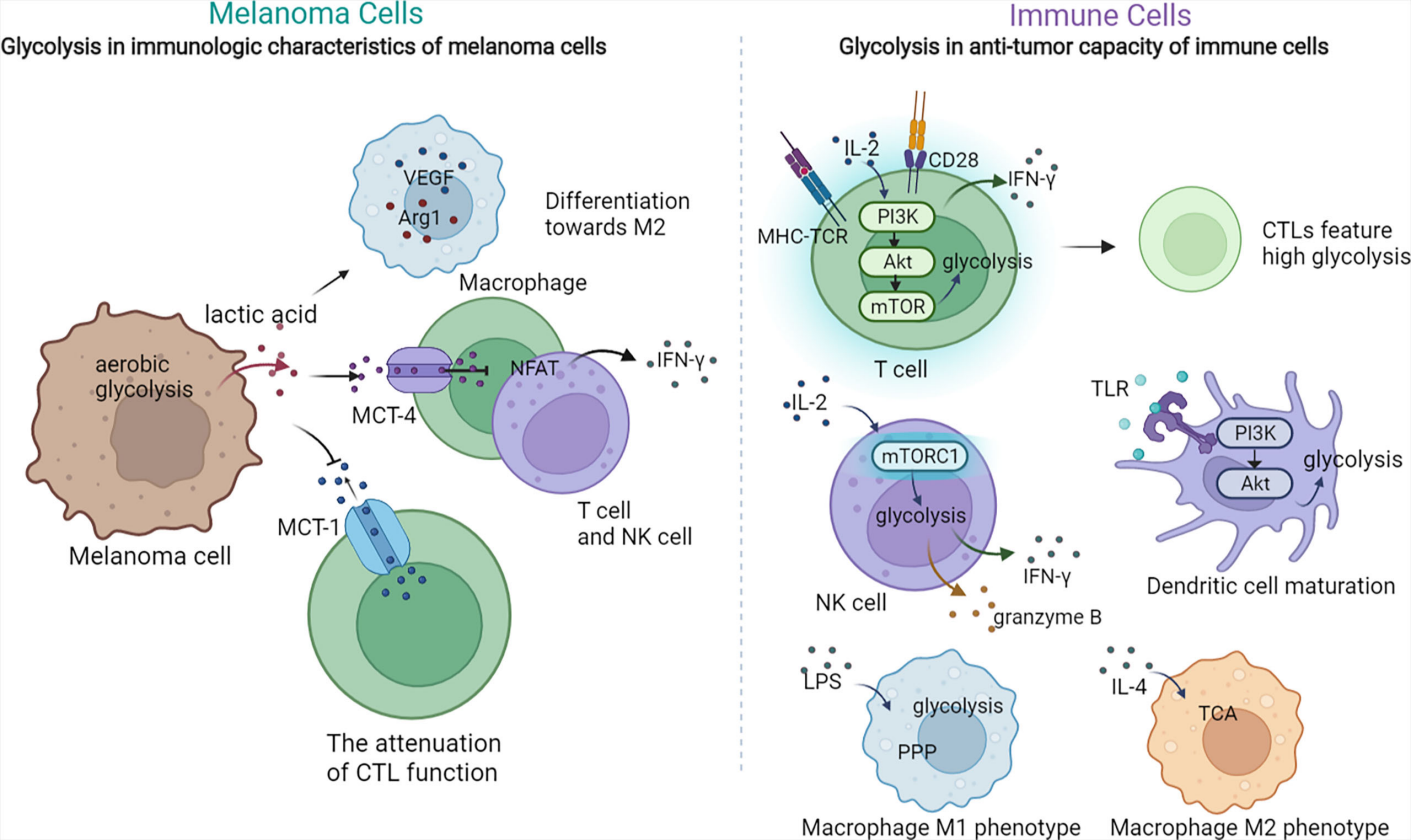
The crosstalk between glycolysis and tumor immunology in melanoma. The dysregulation of glycolysis could exert regulatory multiple effects on the immunologic characteristic of tumor cells and the anti-tumor capacity of immune cells. On one hand, the activated glycolysis in melanoma cells lead to extracellular lactic acid accumulation, which can affect the function of macrophage, T cells and NK cells. On the other, glycolysis in T cells, NK cells and macrophages can also modulate their function and anti-tumor activity.

Aerobic glycolysis in melanoma cells plays a pivotal role in modulating the immunologic features of melanoma through diverse approaches, including extracellular acidification, immune checkpoint expression, expression of immune-related genes, and secretion of cytokines. Increased glycolysis leads to the extracellular accumulation of its end product, lactic acid, which inhibits up to 95% of the capacity of proliferation and secretion of cytokines of immune-infiltrated CTL cells and reduces their activity by 50%, while supporting Treg survival (18, 86). Mechanically, a high concentration of extracellular lactic acid blocks monocarboxylate transporter-1 (MCT-1) on CTLs, which depends on the gradient of cytoplasmic and extracellular lactic acid concentrations to get rid of the lactic acid generated by their own aerobic glycolysis, to accumulate lactic acid in CTLs and eventually attenuate their function (18). In addition, lactic acid-induced extracellular acidification prevents the upregulation of nuclear factor of activated T cells (NFAT) in T and NK cells *via* MCT-4, waning IFN-γ production and consequently attenuating the antitumor functions of T and NK cells (19). Moreover, lactic acid promotes the expression of VEGF and thus the differentiation of tumor-associated macrophages toward the M2 subtype, promoting the growth and metastasis of melanoma cells (87). Apart from causing the extracellular accumulation of lactic acid, glycolysis activates the IFN-α signaling pathway, which accounts for the upregulated expression of PD-L1 and the ultimate immune escape in head and neck squamous cell carcinoma, and the mechanism perhaps remains the same in melanoma (88). In addition, studies on the expression of immune-related genes revealed that the expressions of *CD274*, *PDCD1LG2*, and *TGFB1* are higher in high-glycolysis tumor cells than in low-glycolysis tumor cells (89), indicating that higher glycolysis of melanoma cells attenuates T cells by overexpressing these certain immune inhibitors (90). Particularly, the upregulated expression of *TGFB1* increases the secretion of TGF-β, a significant cytokine mediating immunosuppression of tumor cells (91). For instance, in the TME with a high concentration of TGF-β, the mammalian target of rapamycin (mTOR) signaling pathway in NK cells is inhibited, which results in less production of IFN-γ and impaired antitumor function (92). In sum, aerobic glycolysis in melanoma cells suppresses antitumor immunity within the TME; thus, inhibiting this metabolic pathway may restrain melanoma immune escape (**Figure 2**).

### Glycolysis in the antitumor capacity of immune cells

Immune cells such as T cells, NK cells, M1 cells, and DCs also turn to aerobic glycolysis for nutrition and energy supplementation. Upon activation by the TCR–MHC combination, melanoma-specific CD8^+^T cells upregulate aerobic glycolysis to support their rapid proliferation and anabolic metabolism for building biomass (93, 94). Moreover, the activated PI3K–Akt–mTOR signal pathway upon TCR stimulation enhances glycolysis metabolism in primed CD8^+^T cells and promotes their secretion of IFN-γ, which accentuates the antitumor function of T cells (95). In addition, glycolysis is also related to the differentiation of T cells. While high glycolysis leads to T-cell differentiation toward CTL, a low level of glycolysis is a feature of memory and regulatory T-cell responses (95–97). Nevertheless, in competition with melanoma cells, T cells are deprived of glycolysis, resulting in weakened antitumor function as well as differentiation toward regulatory T cells, which finally triggers melanoma immune evasion (98). As for NK cells, they also depend on glycolysis to support their effector functions upon activation (99, 100). Of note, the level of glycolysis in TAMs depends on their subtype. Upon priming, M1 is characterized with boosted glycolysis and suppressed TCA, whereas M2 features a normal level of TCA with no significant activation of glycolysis (101, 102). In the TME with the shortage of glucose, TAMs differentiate toward the pro-tumoral M2 subtype, which contributes to tumor immune evasion and thereby melanoma progression (103). Moreover, the antigen presentation and inflammatory cytokine production functions of DCs are also impaired in the TME with the shortage supply of glucose, as DCs also rely on glycolysis to exert their antitumor role (104) (**Figure 2**). To sum up, the activation of most tumor-inhibitory immune cells rely on the mTOR-mediated upregulation of glycolysis, and consequently, their functions are attenuated in the TME with scarce glucose or mTOR suppression. Meanwhile, this TME promotes the differentiation toward pro-tumoral immune cells, which ultimately gives rise to melanoma immune escape.

## Mitochondrial oxidative phosphorylation in melanoma immunology

**Figure 3:**
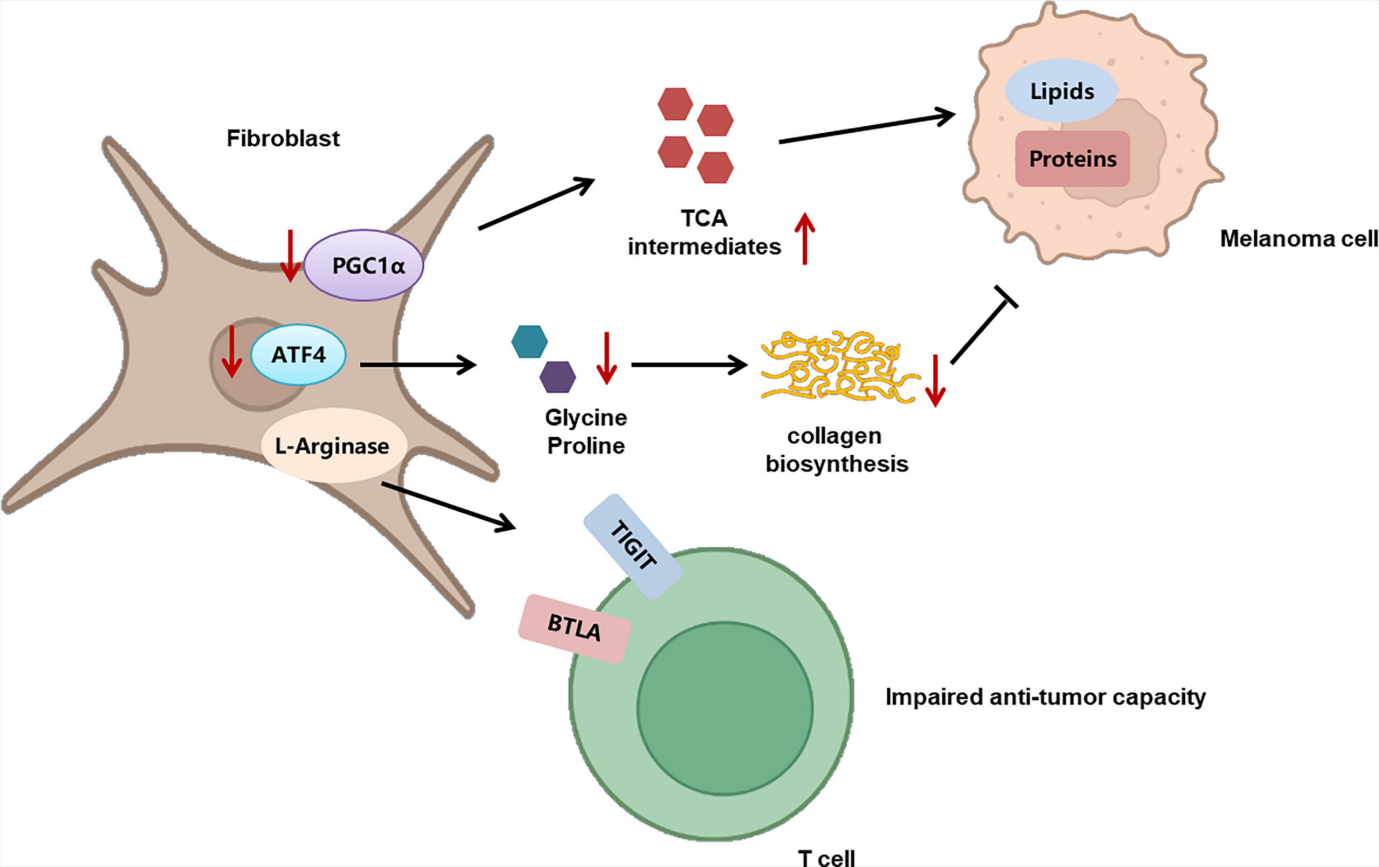
The role of metabolism in CAFs and its implication in melanoma progression. The deficient mitochondrial function induced by PGC1α deficiency triggers the activation of anaplerotic pathways to provide sufficient tricarboxylic acid cycle intermediates, so as to synthesize lipids and proteins to support tumor growth. In addition, CAFs display increased activity of L-arginase, which contributes to TIGIT and BTLA expression on CTLs and impairs the activity of CD8^+^T cells. What’s more, the knockout of ATF4 in fibroblasts leads to defects in collagen biosynthesis and deposition, so as to result in growth delay of melanoma. .

Recently, the role of mitochondrial function in melanoma immunology and the response to immunotherapy have been gradually revealed. An initial proteomics analysis of the clinical samples from advanced-stage melanoma patients undergoing either tumor-infiltrating lymphocyte (TIL)-based or anti-PD-1 immunotherapy has shown that oxidative phosphorylation in general melanoma tissues is significantly higher in responders than in non-responders in both treatments, indicating that mitochondrial function can be used as a potential biomarker for predicting the response to immunotherapy (21). Nevertheless, another study has identified a unique CD8^+^T-cell blood/tumor-shared subpopulation in melanoma patients with high levels of oxidative phosphorylation, which is correlated with immune checkpoint inhibitor (ICI) resistance in melanoma patients. The establishment of a transcriptomic profile reflecting CD8^+^T cells with high oxidative phosphorylation can be effective in discerning responders from non-responders (105). The above two studies seem to display contrary conclusions, which might be due to distinct cellular sources of mitochondria in the TME. Subsequently, the detailed mechanism underlying the effect of mitochondrial function on melanoma immunology has been demonstrated, with particular attention on the antitumor capacity of immune cells. Generally, memory CD8^+^T cells engage oxidative phosphorylation to fulfill their metabolic demands. Potentiating mitochondrial biogenesis in CD8^+^T cells *via* the overexpression of the PGC-1α level can significantly enhance their antitumor immunity in a preclinical melanoma mouse model (22). In line with this, the impairment of mitochondrial function in activated CD8^+^T cells is sufficient to suppress proliferation and upregulate genes linked to the exhaustion of T cells (106). Besides, the dysfunction of the mitochondria is also responsible for the deficiency of the antitumor immunity of T cells during the process of aging, indicating that the mitochondria can build the bridge between aging and its related tumor immunology (107). Furthermore, those tumor-reactive effector/memory cytotoxic T lymphocytes in draining lymph nodes are characterized with increased PGC-1α expression as well as concomitant mitochondrial OXPHOS activation, forwardly indicating the facilitative role of OXPHOS in the antitumor function of T cells (108). Of note, persistent antigenic stimulation conversely inhibits mitochondrial oxidative phosphorylation in activated T cells, leading to their exhaustion and attenuating their suppression on melanoma progression (106). What is more, redox reactions in the mitochondria can result in ROS accumulation, which is also implicated in mediating the effect of the mitochondria on tumor immunology. While elevating mitochondrial ROS (mtROS) promotes the secretion of IFN-γ in TIL *via* a nuclear factor E2-related factor 2 (Nrf2)–(mammalian target of rapamycin complex 1) mTORC1 activation feedback loop and mediate antitumor effectiveness (109), intolerable levels of intrinsic ROS in T cells lead to their exhaustion and impaired antitumor immunity (110). This bifacial effect of ROS may be level-dependent, and quantitative studies of mtROS are in need for a deeper comprehension of its effect on antitumor immunity and future application in immunotherapy. Apart from the role of mitochondria in immune cells, the role of mitochondria in CAFs and the involvement in melanoma pathogenesis have been recently revealed. CAFs are one of the most abundant components of tumor stroma, characterized by a spindle-like morphology and positive for mesenchymal markers such as vimentin (111). In particular, immortalized mouse embryonic fibroblasts (iMEFs) with the knockdown of mitochondrial master regulator PGC1α displayed a decrease in oxidative metabolism and an increase in glycolytic flux. In parallel, PGC1α KO iMEFs helped to form larger and more proliferative primary tumors than WT counterparts and fostered the formation of lung metastasis by B16 melanoma cells (112), which indicated that the metabolic adaption in response to the knockdown of PGC1α and mitochondrial functional deficiency in CAFs played a tumor-facilitative role in melanoma progression. The glucose oxidation and tricarboxylic acid cycle forward flux were reduced after the knockdown of PGC1α, and the anaplerotic pathways were activated to provide sufficient tricarboxylic acid cycle intermediates, so as to synthesize lipids and proteins to support tumor growth. Therefore, PGC1α-mediated mitochondrial function in CAFs acted as a tumor suppressor in melanoma. Targeting oxidative phosphorylation in CAFs might be of high translational potential for melanoma therapy (**Figure 3**).

## The role of amino acid metabolism in melanoma immunology

**Figure 4:**
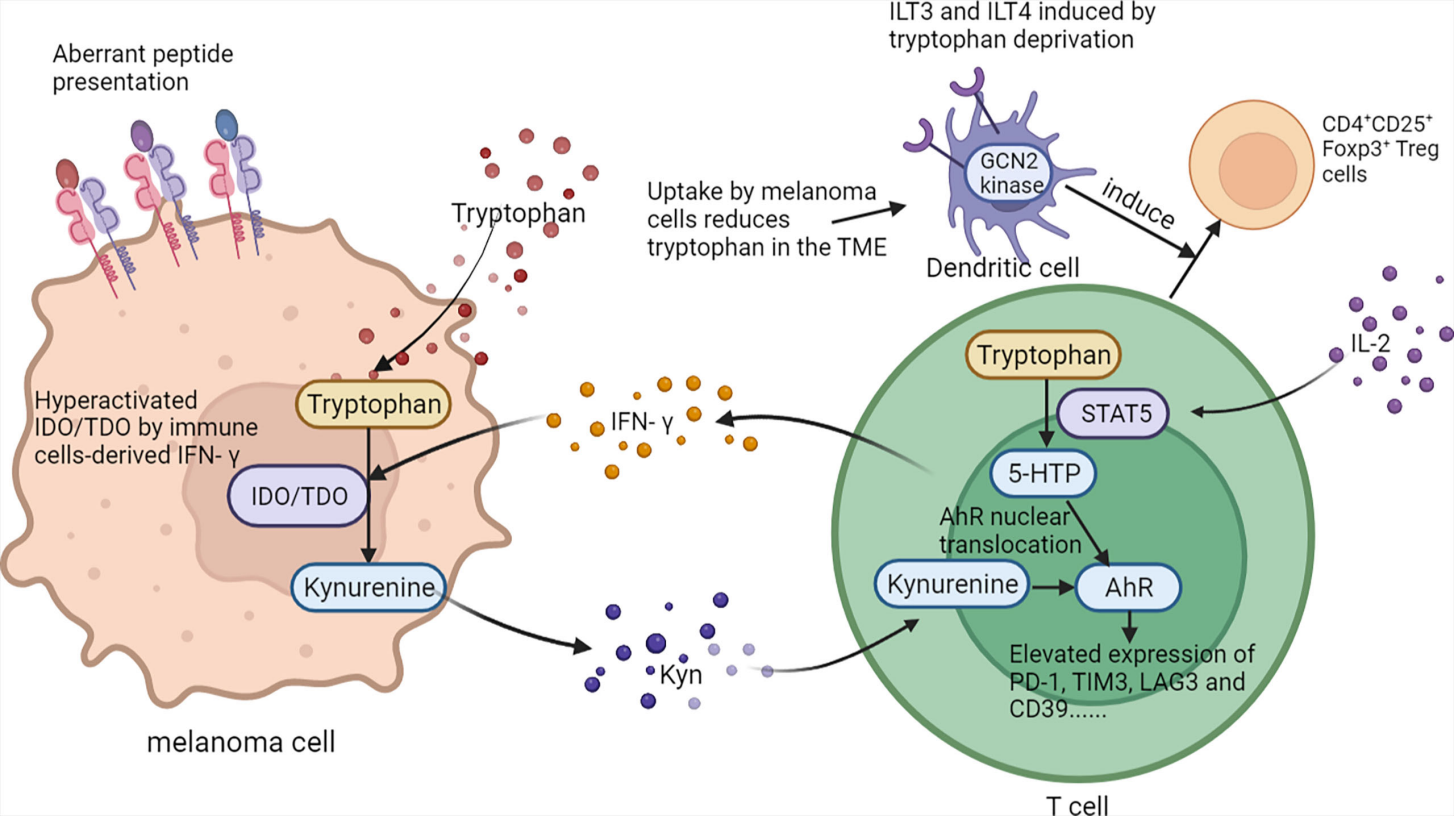
The role of tryptophan metabolism in melanoma immunology. On one hand, abnormally activated IDO1/TDO pathway of tryptophan in melanoma cells contributes to a diversified peptidome landscape and aberrant transframe peptides which could facilitate immune recognition. On the other, tryptophan in TME can affect the function and differentiation of T cells. .

The metabolism of amino acids within the tumor microenvironment is profoundly rewired to support the malignant behaviors of melanoma cells, as well as to mediate the functions of tumor-infiltrating immune cells. An abundant level of amino acid and protein in melanoma cells suppresses antitumor immunity. Rubio-Patino et al. have discovered that a low-protein diet can robustly activate inositol-requiring enzyme-1α (IRE1α) and retinoic acid-inducible gene-1 (RIG1) signaling pathways and induce an unfolded protein response in tumor cells, resulting in augmented cytokine production and increased efficiency of anticancer immune response (113). However, utilizing protein or amino acid restraint to bolster antitumor immunity may not be a feasible tact, for amino acid is fundamental in the antineoplastic functions of immune cells, and its depletion compromises antitumor immunity. Taking CD8^+^T cells as an example, amino acid maintains recombination activating gene (Rag) complex (especially RagD)-mediated mTORC1 translocation to lysosomes and thus supports mTORC1 activity, favoring effective receptor-initiated antitumor immunity (114). Focusing on specific types of amino acid metabolisms, researchers found that cystine, leucine, tryptophan, and arginine metabolisms all play pivotal roles in immune modulations. Melanoma cystine depletion underlies the immunotherapy-related ferroptosis. The treatment with PD-1 antibody increases the secretion of IFN-γ in activated infiltrating CD8^+^T cells, which further suppresses the expression of subunits of glutamate-cystine antiporter system Xc^-^ and leads to restrained tumor cell cystine uptake, potentiated lipid peroxidation, and ultimately ferroptosis (115, 116). In addition to cystine, limited access to leucine (Leu) inhibits the functions of immune cells. For instance, insufficient Leu supply impairs mTORC1 in a RagD-dependent manner and retards the elimination of melanoma cell by T cells. Conversely, supplement of Leu synergizes with the anti-PD-1 antibody to improve the antitumor capacity of T cells *in vivo* (114). Similarly, in Leu-depleted media, mTOR signaling, a sustainer of the initial expression of c-Myc, is also inhibited in NK cells. Consequently, the downregulated c-Myc fails to regulate IL-2/12-stimulated NK cell metabolic reprogramming (including the upregulation of glycolysis) and thus impairs NK cells’ antitumor capacity, which perpetuates tumor progression (117). Compared with cystine and leucine, aberrant tryptophan metabolisms disturb immune responses through more diverse pathways. Deficiency of tryptophan in melanoma cells is found to disturb mRNA translation through ribosomal frameshifting, which results in the presentation of aberrant trans-frame peptides, hence exposing melanoma cells to immune cells (118, 119). Interestingly, this tryptophan depletion in melanoma cells is largely due to RAS activation and could be reversed by pharmacological inhibition of the MAPK pathway, showing that oncogenes somehow paradoxically regulate the immunogenicity of tumor cells *via* the regulation of amino acid metabolism (119). Aside from the influence on the immunological alterations of tumor cells, the abnormally activated indoleamine 2,3-dioxygenase (IDO1)/tryptophan 2,3-dioxygenase (TDO) pathway of tryptophan metabolism alters the immune profile of TME, which is highly related to an unoptimistic prognosis of patients with melanoma. IDO1 and TDO are two main enzymes regulating the first and rate-limiting step of tryptophan catabolism through the kynurenine pathway (120, 121). In melanoma TME, IDO1 and TDO pathways are hyper-activated in both melanoma cells and tumor-infiltrating lymphocytes by immune cell-derived IFN-γ, which reduces tryptophan in TME. This downregulation of tryptophan contributes to a diversified peptidome landscape which may facilitate immune recognition (118). Despite the exposure of tumor-associated antigens, immune cells may not be able to manage antitumor immunity under this condition, for tryptophan (Trp) depletion causes the starvation of cytotoxic T cells and activation of immunosuppressive Tregs (122, 123). In addition, IDO1/TDO activation causes kynurenine (Kyn) accumulation in the TME, which activates the aryl hydrocarbon receptor (AhR), orienting T-cell differentiation into FoxP3^+^ regulatory T cells and the resultant melanoma immune escape (124). Besides, Liu et al. discovered that the transcellular Kyn-aryl-AhR pathway upregulates PD-1 expression in CD8^+^T cells that hampers their activation to eliminate melanoma cells in tumor-bearing mice (125). Moreover, it is found that the IDO1-induced increase of the Kyn/Trp ratio in peripheral blood is a critical predictive biomarker for drug resistance and poor outcome of patients receiving immune checkpoint blockade (126). Considering the tumor-suppressive effect of the IDO pathway, more studies are now focusing on inhibiting it to restore antitumor immunity and enhance the efficacy of immunotherapy. Although the direct inhibition of IDO1 shows uncertainty in treatment outcome, targeting the downstream Trp-Kyn-AhR pathway has been proved to bring a relatively positive outcome (127, 128). In addition to the IDO pathway, signal transducer and activator of transcription 5 (STAT5) activated by a continuously high level of IL-2 induces the conversion of tryptophan to 5-hydroxytryptophan (5-HTP), which activates AhR nuclear translocation and results in an elevated expression of inhibitory receptors such as PD-1, TIM3, LAG3, and CD39, leading to CD8^+^ T-cell exhaustion (129) (**Figure 4**). Apart from cystine, leucine, and tryptophan, L-arginine is also implicated in the regulation of tumor immunology in melanoma. L-Arginine is the precursor of multiple important metabolites, in particular, polyamine and NO, which are of strong immunomodulatory properties. Metabolism of L-arginine through arginase (ARG) and nitric oxide synthase (NOS) enzymes in melanoma reportedly generates diverse metabolic products, which suppresses T-cell function in cancer and causes immune suppression and neovascularization (130, 131). Moreover, arginine metabolism in T cells is crucial for the activation of their antitumor function. Geiger et al. demonstrated that elevated L-arginine content can promote T-cell proliferation and differentiation toward central memory-like T cells with high survivability and antitumor capacity (132). Thus, upregulating CD8^+^ T cells’ extracellular arginine *via* Arg2 deletion or adoptive transfer of Arg2^–/–^ CD8^+^ T results in augmentation of CD8^+^ T-cell activation, effector function, and persistence (133). In addition to amino acid metabolism in tumor cells and immune cells, the alteration of amino acid metabolism in CAFs also participated in melanoma progression. To be specific, compared to normal dermal fibroblasts, melanoma-associated fibroblasts (MAF) displayed increased activity of L-arginase, the selective inhibition of which could neutralize MAF-induced TIGIT and BTLA expression on CTLs and result in the activation of CD8^+^T cells and antitumor immunity (134). What is more, the deficient supply of amino acid glycine and proline induced by the knockout of ATF4 in fibroblasts could lead to significant defects in collagen biosynthesis and deposition and a reduced ability to support angiogenesis, so as to result in a pronounced growth delay of syngeneic melanoma (135). Therefore, the dysregulation of amino acid metabolism in CAFs exerts a versatile role in melanoma pathogenesis *via* the regulation of either tumor immunology or tumor vascularization (**Figure 3**).

To sum up, as substance involved in diverse essential metabolic pathways, the contents of amino acids and related metabolic pathways have great impacts on the immunological characteristics of melanoma cells, immune cells, and CAFs.

## The crosstalk between autophagy and melanoma immunology

Some previous reports have revealed that melanoma autophagy may act as an effective promoter of immune escape. It is observed that hypoxia-induced autophagy in cancer cells leads to STAT3-mediated suppression of the tumor-lysing function of cytotoxic T cells, while inhibition of melanoma autophagy restores the function of CTL through ubiquitin proteasome system and SQSTM1/p62 (136). Recently, genetically targeting the autophagy-related gene *Becn1*/*Beclin1* in B16-F10 malignant melanoma cells increases the infiltration of functional NK cells into melanoma tumors and inhibits their growth. In these beclin 1 (BECN1)-defective tumor cells, the MAPK8/JNK-JUN/c-Jun signaling pathway is activated, leading to the overexpression and secretion of the CCL5 cytokine in TME, which upregulates NK-cell activator NKp46 to facilitate NK cells’ antitumor immunity and is correlated with increased survival of melanoma patients. In addition to BECN1 inhibition, targeting other autophagy-related molecules, such as ATG5 or p62/SQSTM1, or inhibiting melanoma autophagy pharmacologically by chloroquine can similarly induce the expression of CCL5 in melanoma cells and consequently enhance the antitumor capacity of NK cells. This mechanism may serve as a novel therapeutic approach to improve NK-based immunotherapy (137, 138). In addition to NK cells, the inhibition of melanoma autophagy by antimalarial and chemotherapies with anthracycline and doxorubicin helps restore equivalent T-cell infiltration and their intact antitumor function (139). Inhibition of autophagy-related protein phosphatidylinositol 3-kinase catalytic subunit type 3 (PIK3C3/VPS34) in melanoma with a genetic target or pharmacological inhibitors reprograms cold immune checkpoint blockade (ICB)-unresponsive tumors into inflamed immune-infiltrated tumors by recruiting NK and CD8^+^T cells into the tumor bed and as such improves the efficacy of anti-PD-1/PD-L1 immunotherapy (140, 141). Similarly, autophagy in myeloid-derived suppressor cells’ (MDSCs) inhibits the antitumor immune responses, as evidenced by the report that autophagy-deficient monocytic MDSCs result in efficient activation of tumor-specific CD4^+^ T cells and improved antitumor immunity (142). Given the suppressive effect of melanoma cells’ and MDSCs’ autophagy on antitumor functions of immune cells, the combination of autophagy inhibition and immunotherapy can be regarded as a therapeutic approach with probabilities. However, it should also be noted that the autophagic degradation of certain immune checkpoints in melanoma cells is beneficial to the antitumor function of immune cells. Sunitinib, a multitargeted receptor tyrosine kinase (RTK) inhibitor, is found to promote PD-L1 translocation into lysosomes for autophagic degradation by binding to p62 and attenuates the inhibition on T cells’ activation and improves the antitumor function of immune cells. Moreover, sunitinib can synergistically enhance the antitumor effect of the CTLA-4 monoclonal antibody in the preclinical context (143). Of note, compared to the role of autophagy in the regulation melanoma cell immunogenicity, its effects on the antitumor capacity of immune cells are rarely studied. Additional studies are needed to clarify the role of autophagy in the regulation of immune cells’ function in melanoma immunology.

## Lipid metabolism in melanoma immunology

Lipid metabolism is greatly implicated in energy provision, membrane composition, and signal transmission to enable cell proliferation, of which reprogramming is regarded as a hallmark of malignant tumors like melanoma. Previous investigations have revealed that the remodeling of lipid metabolism alters immune features of melanoma cells and tumor-infiltrating immune cells, both of which are decisive for tumor immunology and the treatment outcome of immunotherapy. Specifically, the studies on lipid metabolism in melanoma mainly concentrate on lipogenesis, cholesterol metabolism, and fatty acid oxidation (FAO). Therefore, we comprehensively summarized the regulation of melanoma cell immunogenicity and immune cell characteristics in the TME from the aspects of these crucial lipid metabolism pathways.

### Lipid metabolism in immunologic characteristics of melanoma cells

Some recent reports have highlighted that the dysregulation of lipid metabolism affects the immunologic characteristics of tumor cells to modulate immune evasion and the response to immunotherapy. By profiling the proteome of clinical samples from patients with advanced-stage melanoma undergoing either tumor-infiltrating lymphocyte (TIL)-based or anti-PD1 antibody immunotherapy, the authors discovered that compared to non-responders, responders are demarcated by higher oxidative phosphorylation and lipid metabolism. Potentiated lipid metabolism significantly increases the immunogenicity of melanoma cells by elevating antigen presentation, thereby increasing the sensitivity to T cell-mediated killing both *in vitro* and *in vivo* (21). In addition, the inhibition of *de novo* synthesis of mevalonate (MVA) and cholesterol by lipid-lowering agent statins, including simvastatin, atorvastatin, lovastatin, and fluvastatin, might suppress the expression of PD-L1 on melanoma cells through an AKT- and β-catenin-dependent pathway, which helps to suppress tumor-immune evasion and increase the efficacy of immune checkpoint inhibitor-based cancer therapy in a preclinical tumor model (144). Consistently, another investigation conducted by Xu et al. shows that inhibition of the MVA metabolic pathway in tumor cells elicits type 1 classical dendritic cell (cDC1)-mediated tumor recognition and antigen cross-presentation for antitumor immunity. In particular, the suppression of MVA disrupts prenylation of the small GTPase Rac1 and induces cancer cell actin filament exposure, which can be recognized by c-type lectin domain family 9 member A (CLEC9A) specifically expressed on cDC1s and thus activating infiltrating T cells (145). Of note, the roles of lipogenesis and cholesterol biogenesis in the regulation of antigen presentation seem to be paradoxical, which indicates that different metabolic pathways might exert contrary effects on melanoma cell immunogenicity in the context of lipid metabolism. Furthermore, the status of lipid peroxidation in melanoma cells is decisive for the outcome of immunotherapy. Upon the treatment with the anti-PD-1 antibody, infiltrating CD8^+^T cells would upregulate the secretion of IFN-γ that suppresses the subunit of the glutamate-cystine antiporter Xc^-^ system, which diminishes the uptake of cystine and accumulates lipid peroxidation that induces ferroptosis (116) (**Figure 3**). This regulatory effect is further proved in the context of radiotherapy. IFN-γ derived from immunotherapy-activated CD8^+^T cells and radiotherapy-activated ataxia telangiectasia mutated (ATM) can independently or synergistically suppress the expression of SLC7A11 to reduce cystine uptake, enhance tumor lipid oxidation and ferroptosis, and improve tumor control. Based on this, immunotherapy was found to sensitize tumors to radiotherapy by promoting tumor-cell ferroptosis in a preclinical melanoma mouse model (115). The intervention of lipid metabolism in tumor cells might prominently optimize tumor immune evasion and the response to immunotherapy.

### The role of lipid metabolism in antitumor capacity of immune cells

Compared to the role of lipid metabolism in the regulation of melanoma cell immunogenicity, the effect of lipid metabolism on the antitumor capacity of immune cells was more extensively investigated. Under the circumstance of hypoglycemia and hypoxia in TME, peroxisome proliferator-activated receptor (PPAR)-α signaling and fatty acid catabolism in CD8^+^T cells are activated to preserve the effector functions and confine tumor progression. In a preclinical melanoma model, specifically promoting the fatty acid metabolism can work jointly with the anti-PD-1 antibody to elevate the antitumor function of CD8^+^ T cells in melanoma treatment (146). In accordance, the suppression of fatty acid metabolism due to the accumulation of long-chain fatty acids (LCFAs) in CD8^+^ T cells in pancreatic ductal adenocarcinoma can impair their mitochondrial function and induce lipotoxicity, which dampens CD8^+^T cell-instigated tumor progression (147). Therefore, the positive role of potentiated fatty acid catabolism in the antitumor immunity of CD8^+^T might be highly conserved in different types of cancers. Intriguingly, the senescence of T cells can also be modulated by lipid metabolism. Tumor cells and Treg cells drive the elevated expression of group IVA phospholipase A2 that induces lipid metabolism alteration and senescence in T cells, which is coordinately controlled by MAPK and STAT signals. The inhibition of group IVA phospholipase A2 significantly not only reprograms the lipid metabolism in effector T cells and prevents T cells from senescence *in vitro* but also potentiates the efficacy of immunotherapy in a mouse model of melanoma *in vivo* (148). Apart from T cells, the alteration of fatty acid metabolism also disrupts the function of other immune cells in the TME. For instance, obesity could induce prominent PPAR-driven lipid accumulation in NK cells and cause complete “paralysis” of their cellular metabolism and trafficking, which prevents cytotoxic machinery trafficking to the NK cell-tumor synapse and underlies obesity-related blunted antitumor immunity in tumor (149).

**Figure 5:**
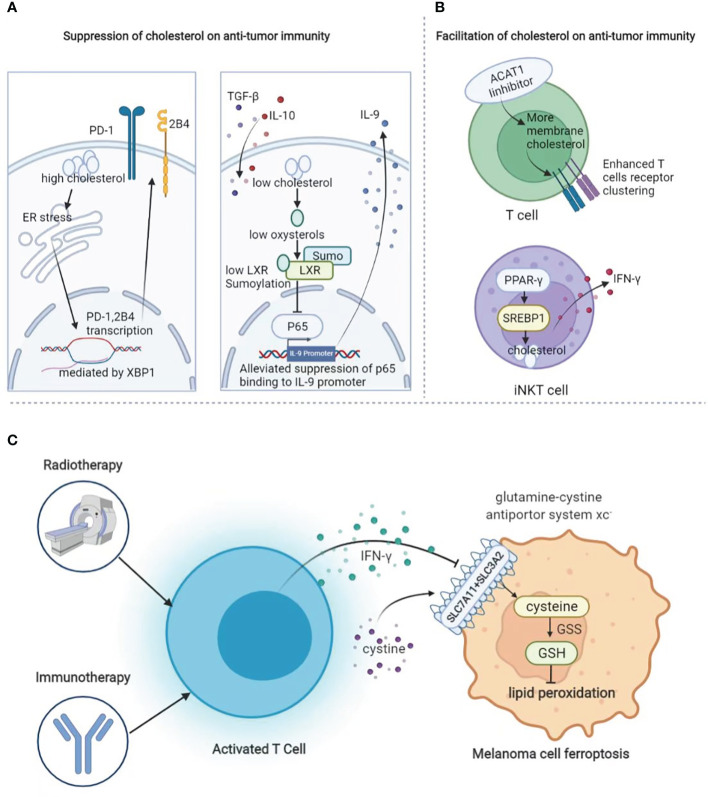
The role of lipid metabolism in melanoma immunology. **(A)** Suppressive role of cholesterol in anti-tumor immunity. Cholesterol promotes the expression of exhaustion-related immune checkpoints in T cells and suppresses the cytotoxic function of Tc9 cells. **(B)** Facilitative role of cholesterol in anti-tumor immunity. Increase of cholesterol *via* targeting at ACAT1 causes enhanced T-cell receptor clustering as well as more efficient formation of the immunological synapse. In addition, increased cholesterol biogenesis *via* PPAR-γ could enhance the transcription of SREBF1 to optimize IFN-γ production. **(C)** During radiotherapy and immunotherapy, activated T cells can secret IFN-γ to suppress the expression of system Xc^-^ to induce lipid peroxidation and thereby ferroptosis.

Similar to fatty acid metabolism, cholesterol biogenesis is also greatly involved in the regulation of the antitumor immunity in TME. Tumor tissues enriched with cholesterol in tumor-infiltrating CD8^+^T cells are positively and progressively associated with upregulated expressions of PD-1, 2B4, TIM-3, and LAG-3. After entering the tumor, CD8^+^T cells harboring high-level cholesterol express increased levels of immune checkpoints and display an exhaustion phenotype. The underlying mechanism is that cholesterol supplement induces endoplasmic reticulum (ER) stress and promotes the transcription of PD-1 and 2B4 *via* x-box binding protein-1 (XBP1) (150). In line with this, the gene profiling of IL-9-secreting (Tc9) cells and classical Tc1 cells suggests that Tc9 cells are of lower cholesterol content, and manipulating cholesterol content in polarizing Tc9 cells can significantly affect IL-9 expression and the antitumor immunity *in vivo* (151). Contrary to these two reports, increasing cholesterol *via* the inhibition of cholesterol esterification by targeting acetyl-CoA acetyltransferase 1 (ACAT1) reportedly potentiates the effector function and cell proliferation of CD8^+^T cells instead of CD4^+^T cells, as the increased plasma membrane cholesterol level enhanced T-cell receptor clustering and efficient formation of the immunological synapse. Either pharmacological or genetic inhibition of ACAT1 in CD8^+^T could control tumor progress better by activating antitumor immunity in mice bearing melanoma (152). Therefore, given the complicated role of cholesterol in CD8^+^T cell-mediated antitumor immunity, it has to be cautious enough to manipulate the process of cholesterol biogenesis and esterification in CD8^+^T cells. In addition to T cells, cholesterol biogenesis also impacts the antitumor immunity of invariant natural killer T (iNKT) cells in TME. PPAR-γ and promyelocytic leukemia zinc finger (PLZF) synergically enhance the transcription of sterol regulatory element binding transcription factor 1 (SREBF1) to promote cholesterol biogenesis, which is required for the optimal IFN-γ production of iNKT cells. The treatment with PPAR-γ agonist pioglitazone promotes IFN-γ production in tumor-infiltrating iNKT cells and helps to prolong the survival of tumor-bearing mice by enhancing the antitumor response (153) (**Figure 5**).

The reprogramming of FAO and lipid peroxidation is also decisive for the antitumor capacity of immune cells, including T cells and Treg cells in TME. Compared to classic Tc1 cells, Tc9 cells exhibit unique lipid metabolic programs. Specifically, Tc9 cell-derived IL-9 can activate STAT3 to increase fatty acid oxidation and mitochondrial activity, which imparts Tc9 cells with reduced lipid peroxidation and the resistance to tumor or ROS-induced ferroptosis. Therefore, lipid peroxidation regulates Tc9-cell longevity and antitumor effects *via* the IL-9-STAT3-fatty acid oxidation pathway (154). Congruently, the upregulated CD36-mediated uptake of fatty acids in CD8^+^T cells can induce lipid peroxidation and ferroptosis, which contributes to reduced cytotoxic cytokine production and impaired antitumor ability. The blockade of CD36 or targeting ferroptosis in CD8^+^T cells acquires greater antitumor efficacy in combination with anti-PD-1 antibodies (155). Taken together, targeting lipid peroxidation might be employed to enhance the T cell-based immunotherapy in melanoma. Extending to this, glutathione peroxidase 4 (GPX4)-mediated lipid peroxidation is also involved in regulating the function of Treg cells. Treg-specific ablation of GPX4 induces robust generation of mitochondrial superoxide and production of IL-1β to facilitate Th17 responses, both of which potentiate antitumor immunity and repress tumor growth in melanoma (156). From this perspective, lipid peroxidation seems to play contrary roles in the regulation of the function in CD8^+^T cells and Treg cells.

In aggregate, different paradigms of metabolic pathways can alter both the immunological characteristics of melanoma cells and the functions of immune cells in TME, ultimately regulating immune escape and the efficacy of immunotherapies in melanoma (**Tables 1**, **2**).

**Table 1:** The crosstalk between metabolism rewiring and melanoma immunology.

Class of metabolism rewiring	Aspect of melanoma immunology	Detailed underlying mechanism		Ref
**Aerobic glycolysis**	**Tumor cell immunologic characteristics**	Extracellular lactic acid accumulation	Extracellular lactic acid blocks monocarboxylate transporter-1 (MCT-1) on CTLs, leads to intracellular accumulation of lactic acid in CTLs, and eventually enervated their function.	(18)
			Extracellular acidification prevents the up-regulation of NFAT in T and NK cells *via* MCT-4, leading to the insufficiency of IFN-γ production.	(19)
			Lactic acid promotes the expressions of VEGF and Arg1 which leads to the differentiation of tumor-associated macrophages towards M2 subtype.	(87)
		Aerobic glycolysis up-regulates PD-L1 expression *via* IFN-α signaling pathway.		(88)
		High-glycolysis melanoma cells upregulates *TGFB1* expression thus increasing the secretion of TGF-β which inhibit mTOR signaling pathway and result in less production of IFN-γ and impaired anti-tumor function.		(89–92)
	**Anti-tumor capacity of immune cells**	T cells	Enhanced glycolysis metabolism in primed CD8^+^T cells promotes their secretion of IFN-γ and IL-2 which accentuates their anti-tumor function.	(95)
			Aerobic glycolysis leads to T-cell differentiation towards CTL, while low level of glycolysis is a feature of memory and regulatory T cell responses.	(95–97)
		NK cells	Glycolysis promotes NK cells’ production of IFNγ and granzyme B, supporting their effector functions upon activation.	(99, 100)
		TAMs	Activation of glycolysis is related with M1 phenotype while shortage of glucose in the TME leads to TAMs’ differentiation towards pro-tumoral M2 subtype.	(101–103)
		DC cells	Glycolysis supports DCs antigen presentation and inflammatory cytokine production functions.	(104)
**Mitochondrial oxidative phosphorylation**	**Anti-tumor capacity of immune cells**	OXPHOS facilitates the anti-tumor immunity of T cells and the inhibition of OXPHOS leads to their exhaustion and attenuates their suppressive function on melanoma progression.		(22) (106–108)
		ROS generated by OXPHOS promotes the TIL secretion of IFN-γ *via* a Nrf2-mTORC1 activation feedback loop, resulting in anti-tumor effectiveness, while intolerable levels of intrinsic ROS in T cells lead to their exhaustion and impaired anti-tumor immunity.		(109, 110)
	**Tumor-regulatory role of CAFs**	The inhibition of mitochondrial function induced by PGC1α knockdown promotes the activation of anaplerotic pathways to provide sufficient tricarboxylic acid cycle intermediates, so as to synthesize lipids and proteins to support tumor growth.		(112)
**Amino acid metabolism**	**Tumor cell immunologic characteristics**	Low protein diet activates IRE1α and RIG1 signaling pathways and thus induce unfolded protein response in tumor cell, resulting in augmented cytokine production and increased efficiency of anticancer immune response.		(113)
		Cystine	Melanoma cystine depletion *via* IFNγ-mediated suppression of glutamate-cystine antiporter system Xc^-^ underlies the immunotherapy-related ferroptosis.	(115, 116)
		Tryptophan	Deficiency of tryptophan in melanoma cells (mediated by MAPK pathway) disturbs mRNA translation through ribosomal frameshifting, which results in presentation of aberrant trans-frame peptides, exposing melanoma cells to immune cells	(118, 119)
			Abnormally activated IDO1/TDO pathway of tryptophan in melanoma cells contributes to a diversified peptidome landscape which facilitates immune recognition.	(120, 121)
		Arginine	L-arginine metabolism through ARG and NOS enzymes in melanoma generates diverse metabolic products which suppresses T cells anti-tumor functions.	(130, 131)
	**Anti-tumor capacity of immune cells**	Leucine	Amino acid (Leu for instance) maintains Rag complex (especially RagD)-mediated mTORC1 translocation to lysosome and thus supports mTORC1 activity in CD8^+^ T cells, leading to effective receptor-initiated antitumor immunity.	(114)
			Leu-depletion inhibits mTOR signaling in NK cells, and thus down-regulates IL-2/12 secretion and hampers NK metabolic reprogramming impairing NK cells’ anti-tumoral capacity.	(117)
		Tryptophan	Try depletion causes the starvation of cytotoxic T cells while activation of immunosuppressive Tregs.	(122, 123)
			IDO/TDO activation-mediated kynurenine (Kyn) accumulation in the TME activates AhR, leading to T cells differentiation into FoxP3^+^ regulatory T cells and thus melanoma immune escape.	(124)
			The transcellular Kyn-AhR pathway up-regulates PD-1 expression on CD8+ T cells.	(125)
			IL-2 induced activation of STAT5 converts tryptophan to 5-HTP, which activates expression of inhibitory receptors such as PD-1, TIM3, LAG3 and CD39, leading to CD8^+^ T cells exhaustion.	(129)
		Arginine	Up-regulated CD8^+^ T cells’ extracellular arginine *via* arginase 2 (Arg2) deletion or adoptive transfer of Arg2^–/–^ CD8^+^ T results in augmentation of CD8^+^ T cell activation, effector function, and persistence.	(133)
	**Tumor-regulatory role of CAFs**	Arginine	The selective inhibition of L-arginase neutralizes MAF-induced TIGIT and BTLA expression on CTLs, and result in the activation of CD8^+^T cells and anti-tumor immunity	(134)
		Glycine and Proline	The deficient supply of glycine and proline induced by ATF4 knockout in CAFs leads to significant defects in collagen biosynthesis and a reduced ability to support angiogenesis, so as to result in pronounced growth delay of melanoma.	(135)
**Autophagy**	**Tumor cell immunologic characteristics**	Hypoxia-induced autophagy in melanoma cells leads to STAT3-mediated suppression of the tumor-lysing function of cytotoxic T cells, while inhibiting melanoma autophagy restores CTLs’ function through ubiquitin proteasome system and SQSTM1/p62 involved down-regulation of phospho-STAT3.		(136)
		Inhibition of melanoma autophagy through targeting at ATG5, p62/SQSTM1 and BECN1 activates MAPK8/JNK-JUN/c-Jun signaling pathway in melanoma cells which up-regulates CCL5 cytokine in the TME and thus enhance NK function *via* activation of NK cell activator NKp46.		(137, 138)
		Inhibition of the autophagy-related protein PIK3C3/VPS34 in melanoma recruits NK and CD8^+^T cells into the tumor bed, and as such improves the efficacy of anti-PD-1/PD-L1 immunotherapy.		(140, 141)
		Sunitinib promotes PD-L1 translocation into lysosome for autophagic degradation by binding to p62, and thus attenuates the inhibition on T cells’ activation and enhances the efficacy of immunotherapy.		(143)
	**Anti-tumor capacity of immune cells**	Autophagy-deficient monocytic MDSCs (M-MDSCs) results in efficient activation of tumor-specific CD4^+^ T cells and improved anti-tumor immunity.		(142)
**Lipid metabolism**	**Tumor cell immunologic characteristics**	Potentiated lipid metabolism increases the immunogenicity of melanoma cells by elevating antigen presentation, thereby increasing sensitivity to T cell mediated killing both *in vitro* and *in vivo*.		(21)
		Mevalonate	Inhibition of *de novo* synthesis of mevalonate suppresses the expression of PD-L1 on melanoma cells through a AKT and β-catenin-dependent pathway	(144)
			Inhibition of MVA elicits type 1 classical dendritic cells (cDC1)-mediated tumor recognition and antigen cross-presentation for anti-tumor immunity	(145)
			MVA suppression disrupts prenylation of the small GTPase Rac1 and induces cancer cell actin filament exposure, which can be recognized by CLEC9A specifically expressed on cDC1s and thus activating infiltrating T cells.	(145)
		IFNγ-mediated suppression of SLC7A11 results in enhanced tumor lipid oxidation and ferroptosis.		(115, 116)
	**Anti-tumor capacity of immune cells**	Fatty acid metabolism	PPAR-α signaling and fatty acid catabolism in CD8^+^T cells are activated in hypoxia TME to preserve the effector functions and slow tumor progression.	(146)
			The suppression of fatty acid metabolism due to the accumulation of LCFAs in CD8^+^ T cells impairs their mitochondrial function and induce lipotoxicity, dampening CD8^+^ T cells and facilitating tumor progression.	(147)
			PPAR-driven lipid accumulation in NK cells causes complete “paralysis” of their cellular metabolism and trafficking and blunts their anti-tumor immunity in tumor.	(149)
		Cholesterol biogenesis	Cholesterol supplement in CD8^+^T cells induces ER stress and promotes the transcription of PD-1 and 2B4 *via* XBP1.	(150)
			Down-regulating cholesterol content induces polarization towards Tc9 cells and enhance IL-9 expression and the anti-tumor immunity *in vivo*.	(151)
			Increase of cholesterol *via* targeting at ACAT1 causes enhanced T-cell receptor clustering as well as more efficient formation of the immunological synapse, leading to potentiated effector function and cell proliferation of CD8^+^T cells but not CD4^+^T cells.	(152)
			Promotion of cholesterol biogenesis *via* PPAR-γ and PLZF synergic enhancement of the transcription of SREBF1 optimizes IFN-γ production of iNKT cells.	(153)
		FAO and lipid peroxidation	Lipid peroxidation regulates Tc9-cell longevity and anti-tumor effects *via* IL-9-STAT3-fatty acid oxidation pathway.	(154)
			CD36-mediated uptake of fatty acids in CD8+T cells induces lipid peroxidation and ferroptosis, which contributes to reduced cytotoxic cytokine production and impaired anti-tumor ability.	(155)
			Robust generation of mitochondrial superoxide and production of IL-1β induced by Treg-specific ablation of GPX4 help to potentiate antitumor immunity and repress tumor growth in melanoma.	(156)

**Table 2:** List of inhibitors of metabolic targets for re-activation of immune cells.

Class of metabolism rewiring	Inhibitors of metabolic targets for re-activation of immune cells	Ref
**Aerobic glycolysis**	**LDH inhibitor Oxamic acid (OA)** Re-activation of cytotoxic T lymphocytes	(18, 86)
	**MCT inhibitor CHC (α-cyano-4-hydroxycinnamate)** Suppression of M2-like phenotype of TAM	(87)
**Mitochondrial oxidative phosphorylation**	**ROS scavenger NAC and VEGFR inhibitor Axitinib** Suppression of the exhaustion of intratumoral T cells	(110)
**Amino acid metabolism**	**Cystine metabolism inhibitors cyst(e)inase and sulfasalazine** Increased efficacy of immunotherapy and radiotherapy by promoting ferroptosis	(115, 116)
	**Tryptophan metabolism IDO1 inhibitor epacadostat** The activation of cytotoxic T cells, the suppression of immunosuppressive Tregs	(121–124)
	**L-arginine metabolism enzymes ARG inhibitor nor-NOHA and CB-1158** Reactivation of CD8^+^ T cells-dependent anti-tumor immunity, including the cytotoxic effect and persistence of CD8^+^T cells	(130–133)
**Autophagy**	**Autophagy inhibitor hydroxychloroquine and chloroquine** Increased tumor-lysing function of cytotoxic T cells, increase of NK cells’ infiltration	(136–138)
	**Autophagy-related protein PIK3C3/VPS34 inhibitors (SB02024 and SAR405)** Increase of the recruitment of NK and CD8^+^T cells	(140, 141)
**Lipid metabolism**	**Mevalonate pathway inhibitor statins** Activation of cDC1-mediated tumor antigen recognition and T cells-dependent anti-tumor immunity	(144, 145)
	**PPARα antagonist GW6471 and PPARδ antagonist GSK3787** The activation of anti-tumor function of NK cells	(149)
	**Cholesterol biogenesis inhibitor simvastatin** The suppression of the exhaustion of CD8^+^T cells and the activation of their anti-tumor capacity The activation of IL-9-producing CD8^+^T (Tc9) cells	(150, 151)
	**Lipid peroxidation-related ferroptosis inhibitor ferrostatin-1** The increase of Tc9-cell longevity and anti-tumor effects	(154, 155)

## Targeting metabolism in melanoma immunology

Based on the mechanistic discoveries of the regulation of melanoma immunology by metabolism, some clinical trials were conducted to verify the effect and safety of the combined therapy with both immune checkpoint blockade and metabolism-targeting drugs. Metformin is a well-known metabolic modulator and inhibitor of mitochondrial function. A previous report firstly documented that metformin treatment could prominently suppress oxygen consumption and result in reduced intra-tumoral hypoxia. Then the combination of metformin and anti-PD-1 antibody is found to improve intra-tumoral T-cell function in a preclinical mouse model (157). In addition, metformin administration elevates mtROS to activate Nrf2, which contributes to mTORC1 activation and IFN-γ production in CD8^+^TILs, thus solidifying the combined treatment effect of metformin and immunotherapy in melanoma (109). Furthermore, favorable treatment-related outcomes (objective response rate (ORR), disease control rate (DCR), median progression-free survival (PFS), and median overall survival (OS)) in patients with melanomas who have received metformin in combination with ICIs were observed in a retrospective study, despite the result dose not reaching significance (158). Of note, another two clinical trials (NCT04114136, NCT03311308) have been conducted to forwardly verify the therapeutic value of the combined complication of both metformin and anti-PD-1 antibody in melanoma and other types of solid tumors.

In addition to mitochondria-regulating agent metformin, drugs targeting amino acid metabolism, in particular, the inhibitors of indoleamine 2,3-dioxygenase 1 (IDO1) enzyme, have been applied in multiple clinical trials in combination with anti-PD-1 immunotherapy in melanoma. A phase 1/2 study of IDO1 inhibitor epacadostat in combination with ipilimumab in patients with unresectable or metastatic melanoma revealed that epacadostat ≤50 mg BID demonstrated clinical and pharmacologic activity and was generally well tolerated (NCT01604889) (159). However, in another study enrolling 706 patients with unresectable stage III or IV melanomas, epacadostat 100 mg twice daily plus pembrolizumab does not improve progression-free survival or overall survival compared with placebo, indicating that the adoption of IDO1 inhibition as a strategy to enhance anti-PD-1 therapy activity in melanoma needs further verification (NCT02752074) (160). Recently, a first-in-class immune-modulatory vaccine (IO102/IO103) against IDO and PD-L1 that targets immunosuppressive cells and tumor cells expressing IDO and/or PD-L1 has been applied in combination with nivolumab to treat patients with melanomas (NCT03047928). As a result, the T-cell influx of peripherally expanded T cells into tumor sites is observed in responsive patients. The clinical efficacy and favorable safety data support further validation in a larger randomized trial to confirm the clinical potential of this therapeutic approach (161). Aside from these clinical trials, there are also some other ongoing trials combining IDO inhibitor and immunotherapy in melanoma, for example, the combination of IDO inhibitor indoximod and ipilimumab/nivolumab/pembrolizumab (NCT02073123). More clinical trials are expected to discover additional promising therapeutic combinations based on the regulation of tumor immunology by metabolism (**Table 3**).

**Table 3:** List of clinical trials combining metabolic drug and immunotherapy in melanoma.

Clinical trial ID		Recruitment Status	Phase	Immunotherapy agent	Metabolic drug	Cancer type
**Mitochondrial inhibitor**	**NCT04114136**	Recruiting	II	Nivolumab& Pembrolizumab	Metformin	Melanoma, NSCLC
	**NCT03311308**	Recruiting	I	Pembrolizumab	Metformin	Advanced Melanoma
**IDO inhibitor**	**NCT01604889**	Terminated	I/II	Ipilimumab	Epacadostat	Melanoma
	**NCT02752074**	Completed	III	Pembrolizumab	Epacadostat	Melanoma
	**NCT03047928**	Recruiting	I/II	Nivolumab	PD-L1/IDO peptide vaccine	Metastatic melanoma
	**NCT02073123**	Completed	I/II	Ipilimumab& Nivolumab& Pembrolizumab	Indoximod	Metastatic Melanoma, Stage III Melanoma, Stage IV Melanoma

To date, there are only limited ongoing clinical trials investigating the therapeutic effect of the combination of immunotherapy agents and metabolism-targeting drugs. Based on the mechanistic discoveries, the nuance of metabolism-targeted strategy between melanoma cells and tumor-infiltrating immune cells might sometimes lead to contradictory effects and therefore dissatisfied treatment outcome. Therefore, more investigations are needed to be conducted to dig out alternative common targets, the intervention of which can simultaneously suppress tumor development and activate antitumor immunity. Apart from the discovery of novel metabolism-related therapeutic targets, optimizing the drug delivery approaches can also be taken into consideration. It has been reported that recombinant adeno-associated virus (rAAV) with a modified tumor-specific promoter can express genes specifically in tumor cells (162). Therefore, it would be possible to more precisely target some specific metabolic enzymes and intermediates in tumor cells *via* rAAV or an alternative delivery system, so as to avoid the contrary effect brought by the nuance of targeting the metabolic pathway between tumor cells and immune cells. What is more, it might be helpful to systematically and comprehensively rewire the metabolism of the TME by simultaneously targeting multiple targets in both tumor cells and immune cells, instead of the intervention of a single metabolic pathway. This strategy based on the notion of systemic biology might bring some new insights in metabolism-targeted melanoma immunotherapy.

## Conclusion and perspective

Melanoma is of rather metabolic heterogeneity and can adaptively rewire its metabolism to meet the energetic demand during progression. The critical pathogenic role of metabolic rewiring in melanoma is supported by accumulative evidence and has been recently extended to be associated with tumor immunology and immunotherapy. In this review, we have comprehensively summarized the cross talk between metabolism and tumor immunology in melanoma and discussed the innovations of targeting metabolism-related regulators combined with immunotherapy from the perspectives of both preclinical mouse model and ongoing human clinical trials. Multiple metabolic paradigms including aerobic glycolysis, mitochondrial oxidative phosphorylation, amino acid metabolism, autophagy, and lipid metabolism are implicated in not only tumor cell biology but also tumor immunology in melanoma. Moreover, the dysregulation of metabolic pathways can affect both the immunologic characteristics of tumor cells and the antitumor capacity of immune cells, and the underlying mechanisms are of rather complexity. Although the current reports have gradually revealed the linkage between cell metabolism and tumor immunology in melanoma, the actual effect of different metabolic paradigms and detailed underlying mechanisms remain far from clear, and even some of the investigations have gained contrary conclusions. For example, while higher oxidative phosphorylation in tumor tissues can be regarded as a potential biomarker for predicting better response to immunotherapy, enhanced mitochondrial function in a unique CD8^+^T-cell blood/tumor-shared subpopulation in melanoma patients is correlated with ICI resistance in melanoma patients. Similarly, under some circumstances, the alteration of one specific metabolic pathway might even lead to contrary effects on tumor immunology. When intra-tumoral hyper-activated aerobic glycolysis induces extracellular lactic acid accumulation that would mitigate the cytotoxic function of CD8^+^T cells, tumor-infiltrating DCs and CD8^+^T cells are also highly dependent on glycolysis to fulfill their antigen presentation function and antitumor capacity, respectively. Therefore, the controversial and paradoxical results obtained from these reports point out the challenge that the intervention of one metabolic paradigm should be based on more credible evidence to precisely identify the subpopulation of patients who might get real benefit. In addition, it should be cautious to avoid the paradoxical effects in case of developing metabolism targeted-based immunotherapy. Additional functional and mechanistic studies are required to elucidate the discrepant conclusion, particularly with the involvement of more innovative technical approaches and the validation in a larger cohort of patients in the future.

Interestingly, it would be also possible to simultaneously obtain the suppression of melanoma progression and the activation of antitumor immunity by intervening one specific metabolic pathway, based on the discoveries of mechanistic studies. For example, the inhibition of autophagy can lead to prominent tumor regression and in parallel induces the potentiation of antitumor immunity *via* the activation of NK cells and CD8^+^T cells, which helps to amplify the antitumor effect of autophagy inhibitors. In addition to this, the intervention of arginine metabolism by targeting arginase not only restrains the proliferation and migration of melanoma cells (163) but also induces prominent augmentation of CD8^+^T-cell activation, effector function, and persistence. These speculations need more experimental proofs and support evidence from future preclinical and clinical studies. What is more important, since there is a strong linkage between cell metabolism and tumor immunology, more and more attention is paid on the combination of immune checkpoint inhibitors with agents targeting metabolic reprogramming (164). At present, there are only few ongoing clinical trials testifying the combinatorial effect of immunotherapy agents and metabolism-targeting drugs like mitochondria-targeting metformin and tryptophan metabolism-related IDO inhibitors. However, the final results of those completed trials are far from satisfactory, which suggests that there are still some key points regarding either the underlying mechanism or the clinical setting waiting to be addressed, and more trials enrolled alternative metabolism-targeting agents should be employed to explore additional potential therapeutic strategies.

It should also be noted that there might be compensated activation of alternative metabolic pathways in case of targeting one specific metabolic paradigm, which would rewire the metabolic network in either tumor cells or immune cells to achieve the biological balance again and mitigate the treatment effect as a result. Actually, previous studies merely emphasize on investigating the relationship among different metabolic pathways and their cross talk in tumor immunology, which poses the obstacle in the path to get a more comprehensive understanding of the metabolic landscape during the process of antitumor immunity and immunotherapy. In the future, the notion of systemic biology should be put into practice in investigation. To simultaneously target multiple metabolic targets in both tumor cells and immune cells, rather than one single metabolic pathway, might be a more practical and useful strategy for metabolism-targeted melanoma immunotherapy.

## Author contributions

WG and CL designed the review. NS, YT, and YC drafted the manuscript and prepared the figures. WG and CL revised the draft. All authors read and approved the final manuscript.

## Funding

This work has received funding from the National Natural Science Foundation of China (No. 81902791), Support Program of Young Talents in Shaanxi Province (No. 20200303), and Young Eagle Project of Fourth Military Medical University (No. 2019cyjhgwn).

## Conflict of interest

The authors declare that the research was conducted in the absence of any commercial or financial relationships that could be construed as a potential conflict of interest.

## Publisher’s note

All claims expressed in this article are solely those of the authors and do not necessarily represent those of their affiliated organizations, or those of the publisher, the editors and the reviewers. Any product that may be evaluated in this article, or claim that may be made by its manufacturer, is not guaranteed or endorsed by the publisher.
